# Lifestyle Factors, Colorectal Tumor Methylation, and Survival Among African Americans and European Americans

**DOI:** 10.1038/s41598-018-27738-x

**Published:** 2018-06-21

**Authors:** Evan L. Busch, Joseph A. Galanko, Robert S. Sandler, Ajay Goel, Temitope O. Keku

**Affiliations:** 1 Channing Division of Network Medicine, Department of Medicine, Brigham and Women’s Hospital and Harvard Medical School, Boston, MA USA; 2 Department of Epidemiology, Harvard T.H. Chan School of Public Health, Boston, MA USA; 3 Department of Medicine, University of North Carolina, Chapel Hill, NC USA; 4 Center for Gastrointestinal Research, Center for Translational Genomics and Oncology, Baylor Scott & White Research Institute, Charles A Sammons Cancer Center, Baylor University Medical Center, Dallas, TX USA

## Abstract

Differences in tumor characteristics might partially account for mortality disparities between African American (AA) and European American (EA) colorectal cancer patients. We evaluated effect modification by race for exposure and patient-outcomes associations with colorectal tumor methylation among 218 AA and 267 EA colorectal cancer cases from the population-based North Carolina Colon Cancer Study. Tumor methylation was assessed in *CACNA1G*, *MLH1*, *NEUROG1*, *RUNX3*, and *SOCS1*. We used logistic regression to assess whether associations between several lifestyle factors—intake of fruits, vegetables, folate, and non-steroidal anti-inflammatory drugs—and tumor methylation were modified by race. Proportional hazards models were used to evaluate whether race modified associations between tumor methylation and time to all-cause mortality. Greater fruit consumption was associated with greater odds of high *NEUROG1* methylation among EA at methylation cut points of 15–35% (maximum OR 3.44, 95% CI 1.66, 7.13) but not among AA. Higher folate intake was associated with lower odds of high *CACNA1G* methylation among EAs but not AAs. Tumor methylation was not associated with all-cause mortality for either group. Race might modify associations between lifestyle factors and colorectal tumor methylation, but in this sample did not appear to modify associations between tumor methylation and all-cause mortality.

## Introduction

Disparities in colorectal cancer (CRC) incidence and mortality between African Americans (AA) and European Americans (EA) have long been noted. Compared to EA, AA have greater age-adjusted CRC incidence per 100,000 individuals (44 AA versus 38 EA) and greater age-adjusted CRC mortality per 100,000 individuals (19 AA versus 14 EA)^1^. These disparities might not be fully explained by differences in screening rates or access to care^2,3^.

A possible contributor to the mortality difference is variation in the biology of the tumors developed by AA and EA. Whether the two sets of tumors have different genetic profiles is unclear. For example, some studies of microsatellite instability (MSI) in CRC tumors have found that, compared to EA, AA have greater incidence of high MSI tumors^4–6^, while another study reported lower AA incidence of high MSI tumors^7^.

Another possible source of CRC tumor variation is differences in epigenetic profiles. Lifestyle and dietary exposures such as fiber intake and smoking have been associated with CRC methylation in humans^8,9^, and exposure to non-steroidal anti-inflammatory drugs (NSAIDs) has been associated with CRC status of the CpG island methylator phenotype (CIMP)^10^. However, it is not clear whether associations between lifestyle and dietary exposures with methylation markers are modified by race, nor whether differences in CRC methylation by race might contribute to survival differences.

We evaluated potential differences by race for two sets of associations: first, between several lifestyle factors—intake of fruits, vegetables, dietary folate, and NSAIDs—and CRC tumor methylation; and second, between tumor methylation and overall survival. Methylation of tumor tissue was assessed using five genes that have been associated with the CIMP phenotype: *CACNA1G*, *MLH1*, *NEUROG1*, *RUNX3*, and *SOCS1*. Hypermethylation of *MLH1* has been associated with development of CRC^11^, while methylation levels of the other markers have been shown to provide sharply-distinguished high- and low-methylation groups of CRC tumors^12^. We hypothesized that associations would be modified by race for both sets of associations. In analyzing the methylation data, we used cut point analyses^13^ to evaluate the etiologic and survivorship implications of different definitions of high versus low methylation for each marker.

## Materials and Methods

### Study Population

Participants were 485 colon adenocarcinoma patients (218 AA, 267 EA) enrolled in the North Carolina Colon Cancer Study (NCCCS)^14,15^ with successful measurement of at least one methylation marker in tumor tissue specimens. Briefly, NCCCS was a population-based case-control study with participants drawn from 33 counties in eastern and central North Carolina. Eligible cases were identified by a rapid ascertainment system set up with the state cancer registry. They were between 40 and 80 years old at the time of enrollment, which began in October 1996 and ended in September 2000. NCCCS controls were excluded from the present analysis because methylation measurements were only performed in tumor tissue. NCCCS collected baseline questionnaire data, including dietary and medication information. The School of Medicine Institutional Review Board at the University of North Carolina at Chapel Hill approved the protocol, and all participants provided informed consent. All research activities were performed in accordance with the ethical standards of the institutional committee and comparable to the 1964 Helsinki declaration and its later amendments.

### Tumor Methylation Measurements

Extraction of genomic DNA from the formalin-fixed, paraffin-embedded tumor tissue collected by NCCCS was described previously^14^. Using the Bisulfite Pyrosequencing process^12,16,17^, methylation of tumor tissue was assessed in the 5 CIMP markers: *CACNA1G*, *MLH1*, *NEUROG1*, *RUNX3*, and *SOCS1*. Briefly, the EZ Methylation Gold Kit (Zymo Research, Orange, CA) was used to modify DNA with sodium-bisulfite. Pyrosequencing was used to analyze the markers. In addition to modified DNA, the polymerase chain reaction included forward primers, biotinylated reverse primers, HotStar Taq polymerase, and water. The primers were as reported previously^16^. For each CIMP marker, the final methylation measurement was recorded as the continuous mean percentage of methylation for all CpG sites evaluated. None of the 5 markers was measured successfully in all 485 participants, thereby reducing the number of participants who could be included in models of any given marker, with the amount of loss varying by marker.

### Covariates

Demographic variables included race (AA or EA), age (continuous), and sex (male or female). Lifestyle factors of interest were self-reported fruit consumption (g/day), vegetable consumption (g/day), folate intake (dietary mcg/day), and NSAID use (number NSAID pills taken/month over the 5 years before study enrollment). Each lifestyle variable was measured as continuous. To assess time to all-cause mortality, the Social Security Death Index was checked for deaths recorded as of 14 August 2010.

### Statistical Analysis

Associations between lifestyle factors and tumor methylation were evaluated using logistic regression models. The independent variables were age, sex, and lifestyle variables. For analysis, lifestyle variables were dichotomized at the 75^th^ percentile based on the distribution in the overall sample (EA and AA combined) and coded as higher intake versus lower intake. The cut point to dichotomize vegetable consumption was 267.5 g/day, fruit consumption was 212.8 g/day, folate intake was 332.2 dietary mcg/day, and 13.0 NSAIDs taken per month. The dependent variable in the model was dichotomous methylation marker status (high versus low). For each marker, we evaluated the impact of varying the definition of high versus low methylation by selecting a series of cut points covering the range of continuous methylation measurements. For any given cut point, we defined high methylation as methylation at or above the cut point and low methylation as methylation below the cut point. The selection of cut points for each marker was as follows (all values are percentages): *CACNA1G*, 5, 10, 15, 20, 25, 30, 35; *MLH1*, 1, 2, 4, 6, 8, 10, 12, 14, 16; *NEUROG1*, 10, 15, 20, 25, 30, 35, 40, 45, 50; *RUNX3*, 3, 6, 9, 12, 15, 18, 21; and *SOCS1*, 3, 6, 9, 12, 15, 18, 21.

Models were stratified by race. To evaluate possible interactions between race and lifestyle variables, we also ran logistic models using AA and EA participants combined. These models included the same independent variables as the stratified models as well as four interaction terms, one for every combination of race and each lifestyle variable.

In addition to the above models in which both lifestyle and methylation variables were dichotomous, we ran several sensitivity analyses. These included logistic models in which the lifestyle variables were continuous and methylation variables were dichotomous. We also ran two sets of linear regression models in which the dependent variable was continuous methylation: one set in which the lifestyle variables were continuous, and one set in which the lifestyle variables were dichotomous. A further sensitivity analysis was dichotomizing each lifestyle variable at its median value in the overall sample, rather than dichotomizing at the 75^th^ percentile.

Associations between each tumor methylation marker and time to all-cause mortality were evaluated using Cox proportional hazards models^18^. Independent variables were sex, age, lifestyle variables dichotomized at the 75^th^ percentile, and dichotomous methylation marker status. The outcome was time from diagnosis to all-cause mortality, censored at 5 years after diagnosis. For each methylation marker, a series of Cox models was run, one for each different cut point to define dichotomous methylation status. We also ran a Cox model for each marker using continuous methylation measurements. As a sensitivity analysis, additional models were run censoring at 10 years after diagnosis.

All analyses described so far examined associations involving a single methylation marker. In addition, we created summary scores to assess methylation profiles across multiple markers. Each of the 5 continuous methylation markers was converted to a dichotomous variable (0 = low methylation, 1 = high methylation). For this purpose, the cut point chosen for each marker was the lowest cut point where associations between lifestyle factors and dichotomous methylation status were observed, or if no such pattern was observed, then we chose the lowest cut point at which the magnitudes of associations between lifestyle factors and dichotomous methylation status diverged between AA and EA. The summary score cut point chosen for each marker was the following: *CACNA1G*, 15%; *NEUROG1*, 10%; *MLH1*, 2%; *RUNX3*, 9%; and *SOCS1*, 3%. We created two summary scores by adding up different combinations of dichotomous markers: all 5 markers (range: 0–5), and a selection of 3 markers (*CACNA1G*, *NEUROG1*, and *RUNX3*; range: 0–3). We then evaluated associations between lifestyle factors and methylation summary scores, as well as associations between methylation summary scores and overall survival, using similar models as were run for single markers.

All modeling results were reported as point estimates and 95% confidence intervals. Models were not adjusted for multiple comparisons because the multiple models in a cut point analysis are not independent^13,19^. To handle missing data, all models were run as complete-case analyses. This meant that methylation summary scores had a missing value if the participant had missing data for any marker included in the score. Analyses were run using SAS 9.4 (SAS Institute, Cary, NC).

### Data Availability

The data are stored in the Center for Gastrointestinal Biology and Disease at the University of North Carolina, Chapel Hill under the direction of author RSS. Investigators wishing to obtain the data can request setting up a Data Use Agreement by contacting RSS (robert_sandler@med.unc.edu).

## Results

EA and AA distributions of lifestyle factors overlapped to a considerable extent (Table 1). The two groups had similar colorectal primary tumor methylation levels for each individual gene and for the methylation summary scores.

**Table 1:** Participant characteristics.

Characteristic	African Americans (n = 218)	European Americans (n = 267)
Age, median (IQR)	63.5 (54.0, 71.0)	67.0 (59.0, 73.0)
Sex, n (%)		
Male	96 (44)	148 (55)
Female	122 (56)	119 (45)
Lifestyle Factors, median (IQR)		
Fruit consumption (g/day)	139.0 (59.1, 233.4)	110.6 (53.5, 201.2)
Vegetable consumption (g/day)	150.0 (108.6, 232.2)	207.8 (147.2, 276.8)
Folate (mcg/day)	231.9 (173.4, 307.0)	259.1 (206.0, 334.9)
NSAIDs (uses/month)	0.0 (0.0, 8.4)	0.0 (0.0, 18.3)
Tumor Gene Methylation (%), median (IQR)		
*CACNA1G*	3.9 (2.5, 10.6)	3.8 (2.5, 8.2)
*MLH1*	1.7 (1.0, 3.6)	1.7 (1.0, 3.0)
*NEUROG1*	20.4 (8.8, 33.7)	20.7 (8.0, 34.6)
*RUNX3*	3.2 (2.1, 5.1)	3.5 (2.2, 6.5)
*SOCS1*	2.8 (2.1, 3.9)	2.9 (2.1, 4.8)
Methylation summary score, All markers (0–5)	2.0 (1.0, 3.0)	2.0 (1.0, 3.0)
Methylation summary score, *CACNA1G*, *NEUROG1*, *RUNX3* (0–3)	1.0 (1.0, 1.0)	1.0 (0.0, 1.0)
Deaths within 5 years of diagnosis, n (%)	70 (32)	66 (25)

IQR = interquartile range, NSAIDs = non-steroidal anti-inflammatory drugs.

For associations between dichotomous lifestyle factors and *CACNA1G* methylation (Table 2), among AA, higher vegetable consumption was associated with greater odds of a high methylation tumor when high methylation was defined by a cut point of 15% (OR = 2.95, 95% CI 1.10, 7.94) but not at other cut points. Vegetable consumption and *CACNA1G* methylation were not associated among EA, and confidence intervals generally overlapped with those for the corresponding AA estimates. Folate intake was not associated with methylation among AA, but among EA, higher folate intake was associated with lower odds of a high methylation tumor when high methylation was defined by cut points ranging from 5–30%. Higher intake of NSAIDs among AA was associated with greater odds of a high methylation tumor at a methylation cut point of 5% (OR = 0.42, 95% CI 0.18, 0.97), but among EA we did not observe an association between NSAIDs intake and *CACNA1G* methylation. Fruits were not associated with *CACNA1G* methylation for either group.

**Table 2:** Associations between lifestyle factors and colorectal primary tumor methylation of *CACNA1G* by race.

Group	*CACNA1GCut Point (%)*	n (%)High	Fruits		Vegetables		Folate		NSAIDs	
			OR	95% CI	OR	95% CI	OR	95% CI	OR	95% CI
AA (n = 190)	5	74 (39)	0.72	0.33, 1.60	1.07	0.46, 2.51	2.16	0.85, 5.52	0.42	0.18, 0.97
	10	48 (25)	0.82	0.35, 1.93	1.54	0.64, 3.71	1.90	0.71, 5.08	0.66	0.27, 1.62
	15	32 (17)	0.46	0.16, 1.31	2.95	1.10, 7.94	1.56	0.48, 5.03	0.96	0.36, 2.58
	20	21 (11)	0.32	0.08, 1.20	2.61	0.79, 8.62	1.35	0.31, 5.77	1.11	0.35, 3.46
	25	12 (6)	0.51	0.10, 2.50	2.99	0.71, 12.61	1.40	0.25, 7.79	2.02	0.54, 7.52
	30	8 (4)	0.90	0.15, 5.33	4.43	0.83, 23.57	1.24	0.18, 8.51	4.01	0.87, 18.38
	35	5 (3)	0.94	0.10, 9.14	7.78	0.88, 68.47	1.15	0.11, 12.05	6.18	0.84, 45.50
EA (n = 233)	5	88 (38)	1.43	0.69, 2.96	1.25	0.65, 2.39	0.30	0.14, 0.66	0.60	0.31, 1.15
	10	52 (22)	0.97	0.42, 2.26	1.18	0.56, 2.48	0.39	0.16, 0.96	0.83	0.40, 1.74
	15	43 (18)	1.18	0.48, 2.90	1.40	0.63, 3.12	0.24	0.08, 0.71	0.83	0.38, 1.85
	20	31 (13)	1.10	0.38, 3.15	1.92	0.77, 4.78	0.13	0.03, 0.59	0.72	0.28, 1.87
	25	29 (12)	1.18	0.41, 3.39	1.98	0.80, 4.95	0.12	0.03, 0.59	0.74	0.28, 1.93
	30	24 (10)	1.42	0.49, 4.15	1.03	0.36, 2.97	0.19	0.04, 0.94	0.82	0.29, 2.27
	35	16 (7)	1.42	0.40, 5.02	0.68	0.17, 2.65	0.16	0.02, 1.39	0.85	0.25, 2.86

N (%) High = number (%) with *CACNA1G* methylation at or above the respective cut point. Each row represents 1 model with a dependent variable of dichotomous methylation status (high versus low) as defined by the given cut point and independent variables of age, sex, and lifestyle variables (fruits, vegetables, folate, NSAIDS). Lifestyle variables were dichotomized (high versus low) at the 75^th^ percentile based on the distribution in the overall sample (AA and EA combined).

AA = African Americans, EA = European Americans, NSAIDs = non-steroidal anti-inflammatory drugs.

For *NEUROG1* methylation (Table 3), fruit consumption was associated with tumor methylation at a cut point of 40% (OR = 0.27, 95% CI 0.08, 0.94) for AA, while for EA, higher fruit consumption was associated with greater odds of a high methylation tumor at methylation cut points of 15–35%. At a methylation cut point of 30%, confidence intervals for EA (OR = 3.44, 95% CI 1.66, 7.13) and AA (OR = 0.67, 95% CI 0.29, 1.56) estimates did not overlap. For both groups, higher vegetable intake was associated (or nearly so) with greater odds of a high methylation tumor at a cut point of 50%, but there was no association at lower methylation cut points. Folate intake was not associated with *NEUROG1* methylation among AA, but higher folate intake among EA was nearly associated with lower odds of a high methylation tumor at methylation cut points of 15–20%. NSAID use was not associated with methylation for either group.

**Table 3:** Associations between lifestyle factors and colorectal primary tumor methylation of *NEUROG1* by race.

Group	*NEUROG1Cut Point (%)*	n (%)High	Fruits		Vegetables		Folate		NSAIDs	
			OR	95% CI	OR	95% CI	OR	95% CI	OR	95% CI
AA (n = 192)	10	140 (73)	1.28	0.52, 3.15	1.51	0.57, 4.03	0.86	0.30, 2.44	1.16	0.50, 2.73
	15	115 (60)	0.95	0.44, 2.05	0.85	0.37, 1.92	1.24	0.50, 3.10	1.02	0.48, 2.14
	20	98 (51)	1.12	0.52, 2.39	0.98	0.43, 2.20	0.95	0.39, 2.34	1.23	0.59, 2.56
	25	76 (40)	0.74	0.34, 1.64	1.01	0.44, 2.34	0.89	0.35, 2.26	1.13	0.54, 2.39
	30	58 (30)	0.67	0.29, 1.56	1.30	0.54, 3.09	1.10	0.42, 2.96	0.73	0.32, 1.65
	35	43 (22)	0.49	0.19, 1.28	1.59	0.62, 4.06	1.19	0.40, 3.54	0.65	0.26, 1.64
	40	27 (14)	0.27	0.08, 0.94	2.26	0.78, 6.50	2.25	0.64, 8.00	0.96	0.34, 2.71
	45	18 (9)	0.40	0.10, 1.55	1.63	0.47, 5.65	2.09	0.50, 8.82	0.61	0.16, 2.32
	50	9 (5)	0.80	0.14, 4.41	5.03	0.98, 25.98	1.35	0.20, 9.16	1.25	0.23, 6.82
EA (n = 234)	10	161 (69)	2.11	0.92, 4.85	1.20	0.61, 2.37	0.58	0.28, 1.22	1.10	0.56, 2.17
	15	141 (60)	2.73	1.24, 6.01	1.44	0.75, 2.76	0.51	0.25, 1.05	1.09	0.58, 2.08
	20	119 (51)	2.51	1.21, 5.21	1.36	0.73, 2.55	0.52	0.25, 1.04	0.80	0.43, 1.49
	25	97 (41)	2.83	1.39, 5.79	1.26	0.67, 2.38	0.62	0.30, 1.28	0.79	0.42, 1.50
	30	75 (32)	3.44	1.66, 7.13	0.96	0.49, 1.88	0.68	0.32, 1.46	0.63	0.31, 1.25
	35	58 (25)	2.68	1.26, 5.70	1.07	0.53, 2.18	0.62	0.28, 1.40	0.65	0.31, 1.36
	40	43 (18)	1.96	0.85, 4.52	1.10	0.50, 2.43	1.00	0.41, 2.41	0.46	0.19, 1.13
	45	27 (12)	1.55	0.52, 4.60	2.08	0.83, 5.23	0.44	0.14, 1.44	0.46	0.15, 1.44
	50	17 (7)	0.88	0.21, 3.69	4.47	1.44, 13.90	0.63	0.16, 2.42	1.01	0.29, 3.51

N (%) High = number (%) with *NEUROG1* methylation at or above the respective cut point. Each row represents 1 model with a dependent variable of dichotomous methylation status (high versus low) as defined by the given cut point and independent variables of age, sex, and lifestyle variables (fruits, vegetables, folate, NSAIDS). Lifestyle variables were dichotomized (high versus low) at the 75^th^ percentile based on the distribution in the overall sample (AA and EA combined).

AA = African Americans, EA = European Americans, NSAIDs = non-steroidal anti-inflammatory drugs.

For *SOCS1* (Table 4), fruit consumption was not associated with tumor methylation for either group. Folate intake was not generally associated with methylation for either group, except for higher folate intake being associated with lower odds of high tumor methylation among EA at a methylation cut point of 3% (OR = 0.30, 95% CI 0.11, 0.80). NSAID use could not be evaluated for AA at several methylation cut points due to zero cells. For methylation cut points where NSAID use was estimable, AA use was not associated with methylation while higher EA use was associated with lower odds of high tumor methylation at cut points of 6% amd 9%. We did not observe an association between vegetable consumption and *SOCS1* methylation for either group.

**Table 4:** Associations between lifestyle factors and colorectal primary tumor methylation of *SOCS1* by race.

Group	*SOCS1Cut Point (%)*	n (%)High	Fruits		Vegetables		Folate		NSAIDs	
			OR	95% CI	OR	95% CI	OR	95% CI	OR	95% CI
AA (n = 117)	3	54 (46)	1.50	0.57, 3.93	0.92	0.32, 2.69	0.89	0.28, 2.84	0.75	0.28, 1.99
	6	13 (11)	1.86	0.32, 10.71	0.25	0.02, 2.47	1.72	0.25, 11.61	1.70	0.36, 8.09
	9	7 (6)	0.90	0.07, 12.20	1.05	0.09, 12.98	1.41	0.05, 39.40	—	—
	12	5 (4)	2.23	0.11, 46.26	1.43	0.09, 22.76	0.75	0.02, 31.84	—	—
	15	4 (3)	2.23	0.11, 46.26	1.43	0.09, 22.76	0.75	0.02, 31.84	—	—
	18	4 (3)	2.23	0.11, 46.26	1.43	0.09, 22.76	0.75	0.02, 31.84	—	—
	21	4 (3)	2.23	0.11, 46.26	1.43	0.09, 22.76	0.75	0.02, 31.84	—	—
EA (n = 149)	3	70 (47)	0.85	0.34, 2.13	1.23	0.53, 2.85	0.30	0.11, 0.80	0.45	0.20, 1.02
	6	32 (21)	1.09	0.38, 3.10	0.56	0.20, 1.51	1.35	0.45, 4.04	0.31	0.10, 0.91
	9	19 (13)	2.80	0.83, 9.48	0.73	0.22, 2.47	0.81	0.20, 3.23	0.20	0.04, 0.97
	12	18 (12)	2.92	0.86, 9.99	0.78	0.23, 2.67	0.90	0.22, 3.63	0.22	0.04, 1.04
	15	13 (9)	2.71	0.61, 11.98	0.25	0.04, 1.47	2.16	0.39, 12.00	0.16	0.02, 1.33
	18	12 (8)	2.19	0.47, 10.29	0.34	0.06, 2.03	1.56	0.25, 9.59	0.19	0.02, 1.61
	21	12 (8)	2.19	0.47, 10.29	0.34	0.06, 2.03	1.56	0.25, 9.59	0.19	0.02, 1.61

N (%) High = number (%) with *SOCS1* methylation at or above the respective cut point. Each row represents 1 model with a dependent variable of dichotomous methylation status (high versus low) as defined by the given cut point and independent variables of age, sex, and lifestyle variables (fruits, vegetables, folate, NSAIDS). Lifestyle variables were dichotomized (high versus low) at the 75^th^ percentile based on the distribution in the overall sample (AA and EA combined).

AA = African Americans, EA = European Americans, NSAIDs = non-steroidal anti-inflammatory drugs.

For both groups, we did not observe any associations between lifestyle factors and colorectal primary tumor methylation of either *MLH1* or *RUNX3*, with a few exceptions. Among AA, higher vegetable consumption was associated with greater odds of high *RUNX3* methylation at methylation cut points of 18–21% (maximum OR = 6.12, 95% CI 1.12, 33.5). Among EA, higher vegetable intake was associated with greater odds of high *MLH1* methylation at a methylation cut point of 4% (OR = 2.10, 95% CI 1.00, 4.42). Among AA, higher vegetable intake was associated with greater odds of high *MLH1* methylation at a methylation cut point of 14% (OR = 6.13, 95% CI 1.02, 36.83). For methylation summary scores, no consistent pattern of association was observed (data not shown).

Sensitivity analyses of associations between lifestyle factors and tumor methylation did not reveal any notable departures from the main analyses, and consequently data for sensitivity analyses are summarized here but not shown. For associations between lifestyle factors and continuous methylation markers, results were generally null but a few associations were observed, mostly consistent with associations observed for dichotomous methylation status. When lifestyle variables were dichotomized at their median values rather than at the 75^th^ percentile, associations that were positive when dichotomized at the 75^th^ percentile tended to become null or to attenuate. Associations between lifestyle factors and methylation summary scores were generally null; we observed a few associations, but these were attenuated compared to anything observed for the strongest associations involving single markers, and with no clear differences between AA and EA. Interaction models including both AA and EA did not detect any main-effects associations between lifestyle factors and methylation.

In terms of patient survival, for EA, high *CACNA1G* methylation was associated with greater hazards of all-cause mortality at methylation cut points of 10% and 20% (Supplementary Table 1a). For both EA and AA, we observed no other associations between colorectal tumor methylation of any marker and time to all-cause mortality (Supplementary Table 1a and b). This lack of association was consistent across sensitivity analyses: methylation single markers and summary scores; continuous and dichotomous methylation variables, including many different cut points for dichotomous variables; extending censoring to 10 years after diagnosis; and for models of the full sample as well as race-stratified models.

## Discussion

Previous research had reported associations between race and CIMP^20^, as well as associations of lifestyle and dietary factors with CIMP^10,20^. This raised the possibility that associations of lifestyle factors with CIMP could be modified by race, and that race-modified differences in colorectal tumor methylation might contribute to survival disparities. In a sample of AA and EA colorectal cancer patients, we found some evidence of possible differences by race of associations between several lifestyle variables and methylation of multiple CIMP markers. The modification by race tended to consist of an association in one group and no association in the other group, rather than associations in opposite directions, with confidence intervals usually, but not always, overlapping between the two groups. For each group and in the overall sample, we observed little evidence of associations between methylation of the evaluated genes and time to all-cause mortality.

Most notably, we observed associations of higher folate intake with lower odds of high methylation of *CACNA1G* among EA, but no associations between folate intake and *CACNA1G* methylation among AA (Tables 2 and 3). There was some evidence that higher vegetable intake was associated with greater odds of high *CACNA1G* methylation among AA, but vegetable consumption was not associated with *CACNA1G* methylation among EA (Table 2). Higher fruit intake was associated with greater odds of high *NEUROG1* methylation among EA, but among AA the association between fruit consumption and *NEUROG1* methylation was either inverse or null (Table 3). These associations were the most robust in our analysis because they persisted across multiple definitions of high versus low gene methylation. We observed other statistically significant associations between lifestyle variables and gene methylation that were significant for only one or two methylation cut points, making these associations more likely to be significant due to chance rather than genuine findings.

The results were highly dependent on variable coding, particularly the cut point used to define dichotomous methylation status (Tables 2–4). Previous studies of continuous biomarkers have demonstrated the many statistical trade-offs between different definitions of high versus low marker level based on different cut points^13,21^. These trade-offs include, among others, the magnitude and precision of an association, proportion of participants defined as having high marker level, sensitivity, specificity, and risk reclassification statistics (e.g. event Net Reclassification Index and non-event Net Reclassification Index)^13,22^. Our results were consistent with these patterns of trade-offs, which should be taken into account when choosing a cut point to dichotomize a continuous variable, whether for etiologic, survivorship, or clinical purposes.

The present analysis had several strengths. First, the investigation was performed in a population-based study with a diverse population. Second, in evaluating how lifestyle factors and race relate to each other in terms of associations with colorectal tumor methylation, the analytic emphasis on effect modification rather than interaction was appropriate, even though this meant focusing on stratified models with less statistical power. Effect modification concerns relationships between two “exposure” variables in which only one is modifiable, whereas interaction is concerned with situations in which both exposures are modifiable^23^. Since lifestyle factors are modifiable but genetic background is not, the most informative etiologic analysis was an evaluation of how race modifies associations between lifestyle factors and colorectal tumor methylation. While we did include interaction terms in etiologic models using the full sample, the primary interest in those models was to assess main effects of race and lifestyle factors with maximum power. As is the case with any evaluation of effect modification, we note that apparent instances of modification apply only to the scale evaluated. In Tables 2–4, this is the odds scale. It is possible that no modification, or different patterns of modification, would be observed in associations on a different scale.

A third strength was our consideration of how colorectal tumor methylation was associated with both cancer risk factors and patient outcomes. Tumor characteristics can be thought of as intermediates on a pathway beginning with disease risk factors and ending in patient outcomes^13^. This meant that evaluation of both etiologic and survivorship components of the path provided a more complete perspective on the role of tumor methylation than would evaluation of only half of the pathway.

Fourth, the analysis was made more informative by our use of cut point analyses to evaluate different definitions of high versus low methylation for each marker in both etiologic and survivorship contexts. While we also evaluated associations using continuous methylation, analyses with continuous markers assume that every 1-unit change is equivalent. The results presented in Tables 2–4 cast doubt on that assumption. In addition, dichotomous or categorical markers are more easily interpreted, especially for clinical purposes in which dichotomous or categorical marker status might correspond to different treatment strategies.

The analysis had several limitations. First, the sample size was not large, even for models using the full sample. However, for stratified models concerned with differences in associations by race, limitations due to sample size might not have been drastic because the main interest was whether effect estimates diverged between AA and EA, not statistical significance. Estimates in the same direction, but with substantial differences in magnitude, could still be relevant for purposes of modification, especially when confidence intervals for the two groups do not overlap. Second, lifestyle variables were assessed by self-report and could have been subject to recall bias. Third, it would have been informative to evaluate associations between tumor methylation and additional survivorship outcomes such as time to colorectal cancer-specific mortality or response to therapy, but no outcomes other than all-cause mortality were available. Fourth, evaluation of methylation of additional genes would have strengthened the analysis, but the 5 genes evaluated were the only ones for which we had data. Lastly, it would have been informative to perform analyses that, in addition to being stratified by race, were further stratified by clinical variables such as tumor stage or histologic type. Due to lack of data on several clinical variables of interest, as well as sample size limitations, we could not adequately address this issue in the present analysis. However, future studies with larger sample sizes and well-characterized clinical data should explore this question.

We found evidence suggesting that associations between some lifestyle variables and colorectal tumor methylation might vary between AA and EA. The differences in associations by race appeared to consist mainly of differences in effect measure magnitude rather than direction. Future research should attempt to replicate our findings in larger samples, as well as incorporating methylation measurements of more genes than the 5 studied here. Proper replication would include analyzing many different cut points to dichotomize methylation levels as shown in Tables 2–4. Future studies should also evaluate associations between tumor methylation and more survivorship outcomes than the one we were limited to here (time to all-cause mortality). Such research will further clarify whether relationships between lifestyle factors, race, and colorectal tumor methylation contribute to disparities in disease incidence and survivorship.

## Electronic supplementary material

Supplementary Results
