# Supplementary material for: Lifestyle Factors, Colorectal Tumor Methylation, and Survival Among African Americans and European Americans
Source: Sci Rep. 2018 Jun 21;8:9470. doi: 10.1038/s41598-018-27738-x (PMC6013500; doi:10.1038/s41598-018-27738-x)
Supplement: Supplementary file 1 — Supplementary Results [file 41598_2018_27738_MOESM1_ESM.pdf]

Lifestyle Factors, Colorectal Tumor Methylation, and Survival Among African Americans and European Americans

Evan L. Busch, Joseph A. Galanko, Robert S. Sandler, Ajay Goel, & Temitope O. Keku

Supplementary Results: Survival Tables

Supplementary Table 1a. Associations of colon tumor methylation of *CACNA1G, RUNX3,* and *SOCS1* with time to all-cause mortality in African Americans and European Americans

Group	*CACNA1G* Cut Point (%)	n (%) High	HR	95% CI	*RUNX3* Cut Point (%)	n (%) High	HR	95% CI	*SOCS1* Cut Point (%)	n (%) High	HR	95% CI
**AA**	**None**	--	0.99	0.96, 1.03	**None**	--	1.01	0.99, 1.03	**None**	--	0.97	0.88, 1.06
	**5**	74 (39)	0.64	0.35, 1.16	**3**	87 (53)	1.28	0.71, 2.32	**3**	54 (46)	0.64	0.31, 1.32
	**10**	48 (25)	0.92	0.49, 1.73	**6**	35 (21)	1.08	0.53, 2.19	**6**	13 (11)	0.61	0.14, 2.65
	**15**	32 (17)	0.93	0.44, 1.95	**9**	17 (10)	1.06	0.41, 2.77	**9**	7 (6)	0.66	0.09, 5.03
	**20**	21 (11)	0.89	0.37, 2.15	**12**	13 (8)	1.39	0.52, 3.76	**12**	5 (4)	0.94	0.12, 7.26
	**25**	12 (6)	0.79	0.24, 2.56	**15**	11 (7)	1.71	0.62, 4.72	**15**	4 (3)	0.94	0.12, 7.26
	**30**	8 (4)	1.26	0.38, 4.18	**18**	10 (6)	1.95	0.69, 5.49	**18**	4 (3)	0.94	0.12, 7.26
	**35**	5 (3)	1.23	0.28, 5.29	**21**	8 (5)	2.89	0.98, 8.51	**21**	4 (3)	0.94	0.12, 7.26
**EA**	**None**	--	1.01	0.99, 1.03	**None**	--	1.01	0.99, 1.03	**None**	--	1.00	0.97, 1.04
	**5**	88 (38)	1.28	0.75, 2.18	**3**	119 (62)	1.04	0.59, 2.05	**3**	70 (47)	1.57	0.80, 3.06
	**10**	52 (22)	1.75	1.01, 3.04	**6**	51 (26)	1.10	0.59, 2.05	**6**	32 (21)	1.62	0.78, 3.38
	**15**	43 (18)	1.55	0.85, 2.82	**9**	42 (22)	0.84	0.42, 1.70	**9**	19 (13)	0.91	0.31, 2.64
	**20**	31 (13)	2.04	1.07, 3.89	**12**	32 (17)	1.01	0.48, 2.11	**12**	18 (12)	0.96	0.33, 2.76
	**25**	29 (12)	1.86	0.96, 3.61	**15**	27 (14)	1.36	0.65, 2.85	**15**	13 (9)	0.63	0.15, 2.68
	**30**	24 (10)	1.40	0.67, 2.91	**18**	26 (13)	1.39	0.66, 2.94	**18**	12 (8)	0.66	0.15, 2.79
	**35**	16 (7)	1.03	0.40, 2.65	**21**	24 (12)	1.27	0.58, 2.77	**21**	12 (8)	0.66	0.15, 2.79

N (%) High=number (%) with methylation at or above the respective cut point. Cut point of "None" means continuous methylation measurements. Each estimate is the methylation marker estimate from a Cox model with a dependent variable of time from colon cancer diagnosis to all-cause mortality (censored at 5 years) and independent variables of methylation marker, age, sex, and lifestyle variables (fruits, vegetables, folate, and non-steroidal anti-inflammatory drugs, with each lifestyle variable dichotomized at the 75th percentile in the distribution of AA and EA combined).

AA=African Americans, EA=European Americans

Supplementary Table 1b. Associations of colon tumor methylation of *MLH1* and *NEUROG1* with time to all-cause mortality in African Americans and European Americans

Group	*MLH1* Cut Point (%)	n (%) High	HR	95% CI	*NEUROG1* Cut Point (%)	n (%) High	HR	95% CI
**AA**	**None**	--	0.94	0.87, 1.02	**None**	--	1.00	0.98, 1.01
	**1**	143 (77)	0.88	0.47, 1.65	**10**	140 (73)	1.04	0.56, 1.94
	**2**	78 (42)	0.79	0.44, 1.40	**15**	115 (60)	1.14	0.66, 1.98
	**4**	42 (23)	0.60	0.28, 1.28	**20**	98 (51)	1.00	0.59, 1.71
	**6**	30 (16)	0.62	0.26, 1.47	**25**	76 (40)	0.89	0.51, 1.56
	**8**	24 (13)	0.51	0.18, 1.44	**30**	58 (30)	0.83	0.45, 1.51
	**10**	20 (11)	0.48	0.15, 1.56	**35**	43 (22)	0.48	0.22, 1.04
	**12**	16 (9)	0.43	0.10, 1.77	**40**	27 (14)	0.76	0.34, 1.72
	**14**	9 (5)	.	.	**45**	18 (9)	0.77	0.30, 1.96
	**16**	8 (4)	.	.	**50**	9 (5)	1.10	0.34, 3.64
**EA**	**None**	--	1.02	0.98, 1.05	**None**	--	1.00	0.98, 1.02
	**1**	175 (77)	0.98	0.52, 1.83	**10**	161 (69)	0.79	0.45, 1.39
	**2**	106 (46)	0.90	0.53, 1.51	**15**	141 (60)	1.04	0.60, 1.80
	**4**	48 (21)	1.11	0.59, 2.06	**20**	119 (51)	1.13	0.67, 1.93
	**6**	28 (12)	1.13	0.55, 2.35	**25**	97 (41)	1.17	0.68, 1.99
	**8**	26 (11)	1.34	0.64, 2.80	**30**	75 (32)	0.98	0.55, 1.75
	**10**	20 (9)	1.28	0.57, 2.91	**35**	58 (25)	0.96	0.51, 1.81
	**12**	16 (7)	1.44	0.60, 3.47	**40**	43 (18)	1.19	0.61, 2.33
	**14**	13 (6)	1.60	0.62, 4.09	**45**	27 (12)	1.28	0.57, 2.85
	**16**	12 (5)	1.70	0.66, 4.37	**50**	17 (7)	1.38	0.54, 3.49

N (%) High=number (%) with methylation at or above the respective cut point. Cut point of “None” means continuous methylation measurements. Each estimate is the methylation marker estimate from a Cox model with a dependent variable of time from colon cancer diagnosis to all-cause mortality (censored at 5 years) and independent variables of methylation marker, age, sex, and lifestyle variables (fruits, vegetables, folate, and non-steroidal anti-inflammatory drugs, with each lifestyle variable dichotomized at the 75th percentile in the distribution of AA and EA combined).

AA=African Americans, EA=European Americans
